# Euglycaemic ketoacidosis in pregnant women with COVID-19: two case reports

**DOI:** 10.1186/s12884-021-03928-w

**Published:** 2021-06-16

**Authors:** Margaret Pikovsky, Min Yi Tan, Amanda Ahmed, Lynne Sykes, Rochan Agha-Jaffar, Christina K. H. Yu

**Affiliations:** 1 St Mary’s Hospital, Obstetrics Department, Imperial College NHS Trust, Praed Street, London, W1 2NY UK; 2 March of Dimes Prematurity Research Centre, Department of Metabolism, Digestion and Reproduction, Imperial College London, London, W12 0HS UK; 3 Endocrinology Department, St Mary’s Hospital, Imperial College Healthcare NHS Trust, Praed Street, London, W1 2NY UK

**Keywords:** COVID-19, Pregnancy, Ketoacidosis, Euglycaemic, EKA

## Abstract

**Background:**

Euglycaemic ketoacidosis (EKA) is an infrequent but serious condition which usually follows a period of starvation, severe vomiting or illness in individuals with or without diabetes. Ketoacidosis is associated with materno-fetal morbidity and mortality necessitating prompt diagnosis and management. Physiological increases in insulin resistance render pregnancy a diabetogenic state with increased susceptibility to ketosis. COVID-19 is associated with worse clinical outcomes in patients with diabetes and is an independent risk factor for ketoacidosis in normoglycaemic individuals.

**Case presentations:**

We describe two cases of SARS-CoV-2 positive pregnant women presenting with normoglycaemic metabolic ketoacidosis. Both cases were associated with maternal and fetal compromise, requiring aggressive fluid and insulin resuscitation and early delivery.

**Conclusion:**

We discuss possible physiology and propose a management strategy for euglycaemic ketoacidosis in pregnancy.

## Background

In late 2019 a novel coronavirus (SARS-CoV-2) emerged in Wuhan, China and rapidly developed into a global pandemic [1]. Due to the physiological and immunological changes associated with pregnancy, women are particularly vulnerable to respiratory pathogens during the antenatal and peripartum period [2]. Indeed, COVID-19 in pregnancy is associated with more severe illness, higher rates of admission to intensive care, and higher mortality than in the non-pregnant population [3, 4]. Additional challenges, such as hypercoagulability and increased thromboembolic risk, have also been recognised [5].

Pre-existing diabetes mellitus is associated with increased COVID-19 disease severity and mortality [6, 7]. Diabetic ketoacidosis (DKA) has been recognised as a complication of COVID-19 infection and a poor prognostic sign [8, 9]. COVID-19 may precipitate ketosis and ketoacidosis without diabetes mellitus with ketosis independently associated with increased acute respiratory distress syndrome (ARDS), increased hospital stay and mortality in younger patients [10].

During pregnancy the presence of placental derived hormones directly impairs insulin sensitivity and confers a propensity to ketosis [11]. Hence, periods of increased stress secondary to intercurrent illness or prolonged starvation may predispose the pregnant woman to severe metabolic acidosis even in the setting of a normal blood glucose. We present two such cases and demonstrate how COVID-19 can exacerbate metabolic dysregulation, resulting in maternal and fetal compromise. We also illustrate how prompt recognition and treatment of metabolic acidosis in this context can be crucial for positive maternal and fetal outcomes [12].

## Case presentations

### Case one

A 34-year-old, South Asian para 3 with a BMI of 25 was brought in by ambulance at 35 weeks gestation due to breathlessness following a positive SARS-CoV-2 test 6 days earlier. Further symptomatology included nausea. Fetal movements were normal. Past medical history included Type 2 diabetes managed with metformin and insulin (baseline HbA1c 102 mmol/mol), and mild asthma. She had omitted her diabetes therapy due to reduced oral intake but reported satisfactory blood glucose readings in the preceding days. On admission the following was noted: maternal tachycardia, tachypnoea, 95% saturations on air and accessory muscle use with normal vesicular breath sounds (Table 1 for observations and blood results). The fetal heart rate measured 180 bpm with reduced variability and recurrent deep unprovoked decelerations. The computerised cardiotocograph (CTG) showed a pre-terminal trace with a short-term variation of 2.2 m/sec. Venous blood gas analysis showed a severe metabolic acidosis (pH 6.87, pCO_2_ 4.5 kPa, HCO_3_ -6.2 mmol/L, base excess -27 mmol/L, with an increased anion gap (21 mEq./L). Lactate was 2.2 mmol/L, capillary glucose 4.4 mmol/L and capillary ketones 5.2 mmol/L: CRP was 49 mg/L with a lymphopenia of 0.7 × 10^9^/L. COVID-19 prognosticators showed normal lactate dehydrogenase (LDH), troponin T and ferritin level. An intravenous insulin infusion was commenced with aggressive fluid and potassium replacement. There was a marked improvement 8 h later (pH 7.33, pCO2 1.9 kPa, base excess -18 mmol/L, glucose 8.8 mmol/L, blood ketones 2.8 mmol/L) and an emergency caesarean section was performed under regional anaesthesia. A 3.1 kg live infant was delivered in good condition, with Apgars of 8/9/10 at 1, 5 and 10 min respectively. The cord gases showed a pHA 7.06 BE -17; pHV 6.95, base excess-16.9 mmol/L which resolved following delivery. The neonate was admitted at 12 h of age to the special care baby unit with hypoglycaemia (blood glucose level 1.1 mmol/l) and managed with bolus dextrose and maintenance fluids according to the neonatal hypoglycaemia protocol.

**Table 1:** Observations and blood results for Case 1 and Case 2 at the time of initial presentation. Initial results from case 1 were from a venous sample taken in room air, whereas initial blood was taken from case 2 from an arterial sample in room air

**Parameter**	**Case 1**	**Case 2**	**Normal Ranges**
Respiratory rate (br/min)	33	44	14–20
Oxygen saturation in room air (%)	98	94	> 96
Heart rate (bpm)	122	90	60–110
Blood pressure (mmHG)	133/85	118/72	< 140/90
Temperature (C)	36.6	36.6	< 37.5
pH	6.87	7.25	7.35–7.45
pCO_2_ (kPA)	4.4	2.2	4.7–6.0
pO_2_ (kPA)	3.1	30.3	10–13 * arterial
Base excess (mmol/L)	-27.2	-20.2	-2- + 2
Bicarbonate (mmol/L)	6.2	7.1	16–28
Capillary Ketones (mmol/L)	5.2	6.8	< 0.6
Capillary Glucose (mmol/L)	4.4	4.2	4–7
Haemoglobin (g/L)	144	118	114–150
Haematocrit (L/L)	0.424	0.353	0.350–0.450
Platelets (× 10^9^/L)	222	337	135–400
White cell count (× 10^9^/L)	5.1	8.1	4.2–11.2
Lymphocytes (× 10^9^/L)	0.7	0.6	1.1–3.6
Sodium (mmol/L)	136	135	133–146
Potassium (mmol/L)	6.2	3.8	3.5–5.3
Chloride (mmol/L)	109	110	95–108
Urea (mmol/L)	3.9	1.1	2.5–7.8
Creatinine (umol/L)	78	59	55–110
C-reactive protein (mg/L)	49	102	0–5
Lactate (mmol/l)	2.2	0.8	< 2.0
Ferritin (ng/ml)	44	29	10–120
Lactate dehydrogenase (unit/L)	229	279	125–243
Troponin T (ng/L)	< 5	< 0.5	0–15

Arterial gas sampling taken when Case 2 on 60% Oxygen

Following delivery, the patient was recommenced on her pre-pregnancy insulin regime (metformin and long-acting insulin). She desaturated to 88% on day 1 post-delivery and was admitted to the medical ward due to her increasing oxygen requirements. A CTPA demonstrated bilateral peri-bronchovascular and peripheral consolidation, compatible with COVID-19 pneumonitis affecting 50–75% of lung parenchyma. Bilateral pleural effusions and subcutaneous oedema of the soft tissue was also noted. She was subsequently commenced on both dexamethasone and remdesevir, in view of significant COVID-19 lung changes. Baby and mother were reunited on day 10, and they were discharged home on day 12. The mother continued with prophylactic low molecular weight heparinfor 6 weeks.

### Case two

A 34-year-old Caucasian woman, para 1, with a BMI of 28 presented at 36^+3^ weeks gestation with a 3-day history of -shortness of breath, pleuritic chest pain, lethargy and a 2-week history of anorexia. She had no significant medical background, and no personal history of diabetes. On admission she was unwell, tachycardic and tachypnoeic (respiratory rate of 40 bpm), saturating 98% on 15L via non-rebreather (see Table 1 for observations and blood results). Initial arterial blood gas illustrated a raised anion gap metabolic acidosis (pH7.25, pCO2 2.2 kPa, HCO3 -7.1 mmol/L, base excess -20.2 mmol/L, lactate 0.8 mmol/L). The capillary glucose measured 4.2 mmol/L and capillary ketones 6.8 mmol/L: CRP measured 102 mg/L, and a lymphopenia of 0.6 × 109/L. COVID-19 prognosticators showed a high LDH (279 units/L), normal troponin T and ferritin level. Fetal movements and fetal heart rate were normal. She was initially treated with intravenous 10% dextrose for suspected starvation ketoacidosis. COVID-19 infection was confirmed and the CTPA demonstrated bilateral peripheral and basal ground glass appearance and peripheral consolidation, with extent of abnormality estimated at between 26–50% of lung parenchyma. Intravenous hydrocortisone was started. An intravenous insulin infusion was set up, with fluid resuscitation and electrolyte replacement. Prophylactic dose low molecular weight heparin was administered daily throughout admission. Maternal metabolic acidosis resolved over 24 h, but the computerised CTG failed to meet Dawes-Redman criteria on multiple occasions. Her delivery was expedited by caesarean section under regional anaesthesia. A live female infant was delivered in good condition weighing 3.28 kg. Apgar scores and cord gases were normal. The mother was discharged home on day 5 with a course of oral antibiotics, oral steroids and 6 weeks of prophylactic low molecular weight heparin.

## Discussion and conclusion

Ketoacidosis is a severe metabolic disorder mostly seen in diabetes. COVID‐19 infection is associated with ketosis or ketoacidosis in 6.4% of patients requiring hospitalisation, with 35% of these patients affected by diabetes [10]. Data on pregnancy-related euglycaemic ketoacidosis in patients with concomitant COVID-19 infection is sparse, with only two cases so far [12, 13].

Physiological alterations to hormonal and anatomical changes in pregnancy result in respiratory alkalosis with compensatory renal excretion of bicarbonate. The reduction in bicarbonate reduces the buffering capacity of the acid–base balance, thus predisposing pregnant woman to metabolic acidosis.

**Fig. 1:**
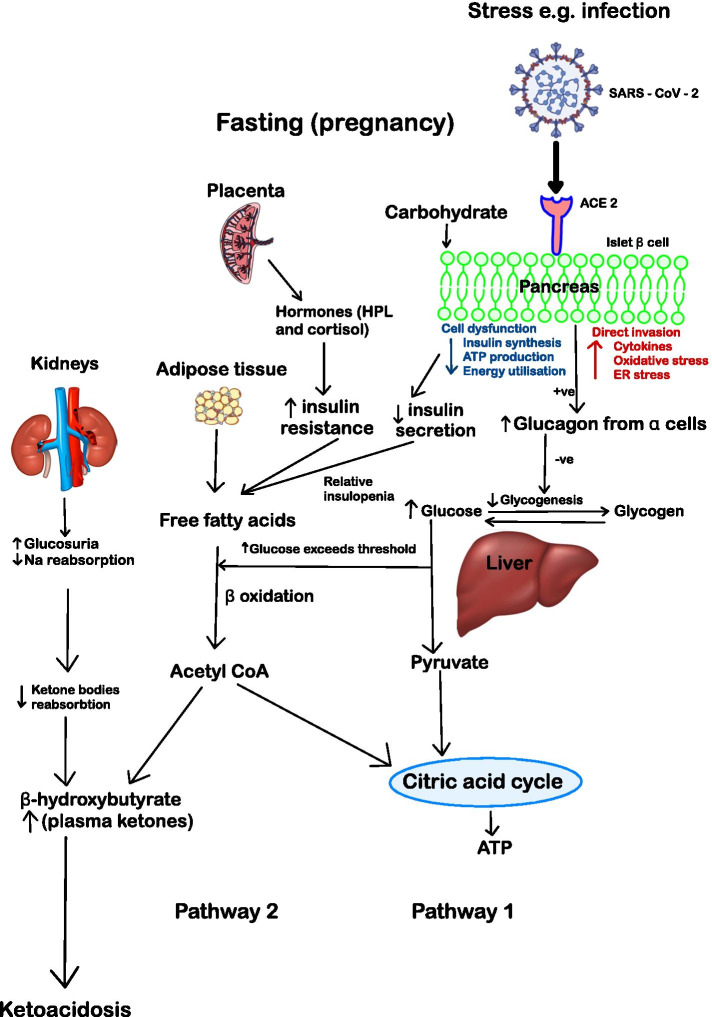
Metabolic changes in the fasting pregnant state. Pathway 1 describes the situation of relative glucose metabolism in the context of acute stress as it is observed in SARS-CoV-2 infection. Pathway 2 describes the situation of glucose metabolism in the context of insulopenia with subsequent fatty acid metabolism and ketosis

Pregnancy is a diabetogenic state with increased insulin resistance, enhanced lipolysis, elevated free fatty acids and increased ketogenesis. Pregnant women are more susceptible to DKA compared to non-pregnant women with diabetes (8.9% vs 3.1%, respectively) [14]. Blood glucose levels tend to be lower in DKA in pregnancy than in the non-pregnant state due to the increased glucose uptake by the fetoplacental unit and a reduction in glycogenolysis and hepatic gluconeogenesis [15]. Hence, increased stress secondary to intercurrent illness or prolonged starvation may predispose the pregnant woman to severe metabolic acidosis with a normal blood glucose (Fig. 1).

The absence of hyperglycaemia in raised anion gap metabolic acidosis often creates a diagnostic challenge. Euglycaemic diabetic ketoacidosis (EDKA) is a biochemical triad of increased anion gap metabolic acidosis, ketosis and normoglycaemia. Starvation ketoacidosis refers to the metabolic acidosis following a period of starvation. Starvation ketoacidosis has been reported rarely in the third trimester due to an exaggerated response to fasting [11, 16, 17].

Both EDKA and starvation ketoacidosis are usually precipitated by a period of fasting or malnutrition which may be triggered by illness. Regardless of the aetiology, severe metabolic acidosis during pregnancy is a serious condition with increased maternal and neonatal morbidity and mortality.

Patients with COVID-19 with diabetes have worse clinical outcomes [15, 18, 19] due to substantial insulin resistance and new onset diabetes in relation with COVID-19 [20–22].

SARS-CoV-2 is a single strand RNA virus which binds to angiotensin converting enzyme 2 (ACE2). ACE2 is a key enzyme in the renin–angiotensin–aldosterone system (RAAS) and catalyses the conversion of angiotensin II to angiotensin. ACE2 is found in the lungs and pancreas and can serve as a cellular entry point for SARS-CoV-2. The direct pancreatic islet cell injury and downregulation of ACE2 expression is thought to lead to unopposed angiotensin II and reduced insulin production: ACE2-mediated dysfunction of the sodium dependent glucose transporter (SGLT1 and/or SLC5A1) at the intestinal epithelium is believed to further exacerbate glycaemic dysregulation [23–25].

The relationship between SARS-CoV-2 and the RAAS also complicates fluid management. Angiotensin II enhances pulmonary vascular permeability and excessive fluid resuscitation can worsen any pre-existing respiratory compromise. Angiotensin II additionally stimulates aldosterone leading to potassium loss; therefore, potassium replacement is needed especially when insulin treatment is required [20, 26].

**Fig. 2:**
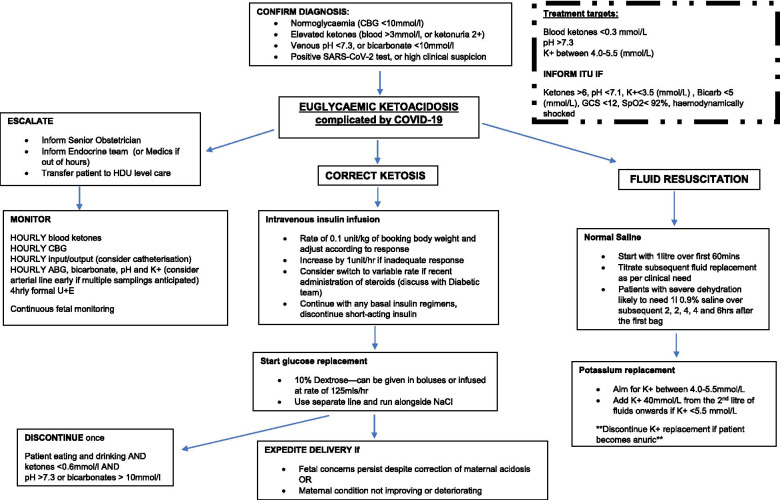
Flowchart depicting proposed management, including monitoring requirements, fluid resuscitation and insulin administration protocols, and escalation to critical care

We propose a management strategy for COVID-19 associated euglycaemic ketoacidosis in pregnancy (Fig. 2), adapted from existing protocols of treatment for hyperglycaemic DKA in non-pregnant and pregnant patients [27, 28]. Criteria for the proposed management pathway are capillary ketones (> 3 mmol/L, or 2 + ketonuria), metabolic acidosis (venous pH < 7.3, or bicarbonate < 10 mmol/L), and normoglycaemia (CBG < 10 mmol/L [16]; with either a positive SARS-CoV-2 test or high clinical suspicion. Prompt escalation to a senior obstetrician, endocrine / medical colleagues and critical care is recommended as patients will require high dependency care. As with the management of hyperglycaemic DKA, hourly monitoring of maternal ketones, glucose, pH and electrolytes are essential to accurately adjust therapy. Strict fluid balance should be recorded and catheterisation considered. The primary goals of management are fluid resuscitation, insulin administration, and electrolyte correction.

Fluid requirements are lower in pregnancy and dehydration and vomiting contribute to the volume depletion characteristic of ketoacidosis. Initial management include 1 L of normal saline over 1 h. To mitigate for any preceding periods of starvation (as in the examples of our two case studies) and maintain normoglycaemia if insulin is administered, addition of dextrose should be considered after the first bag of saline. The use of 10% glucose is preferable to the 5% preparations as smaller volumes can be administered and the rapid metabolism of 5% dextrose means that the solution becomes hypotonic and does not remain in the intravascular space, limiting its use in fluid resuscitation. To avoid pulmonary sequelae due to SARS-CoV-2 and RAAS interplay, subsequent fluid replacement should be titrated according to clinical parameters such as volume status, urine output and biochemical parameters. Patients with a significant period of starvation, or who have other risk factors for thiamine deficiency (e.g., history of severe hyperemesis in pregnancy, ‘abdominal’ COVID-19) are at potential risk of Wernicke’s: intravenous thiamine should therefore be commenced prior to any dextrose administration.

COVID-19 infection can also disrupt metabolic control through reduced insulin production, which can be exacerbated by increased insulin resistance in pregnancy thus increasing vulnerability to ketoacidosis. In addition to fluid resuscitation, initiation of intravenous insulin therapy to suppress ketosis is recommended This is all the more pertinent if preterm delivery is imminent and the patient has received steroids [28].

Potassium correction is required if levels are < 5.5 mmol/L as insulin leads to an intracellular shift of potassium, which is further exacerbated by COVID-19 infection. Potassium replacement should be discontinued if the patient becomes anuric. Maternal and fetal hyponatraemia will be prevented by using saline-containing fluids for volume replacement. Bicarbonate administration is not recommended unless under the guidance of critical care colleagues, as while it may help correct acidosis it can also lead to decreased fetal oxygen delivery, paradoxical cerebral acidosis and augmented ketogenesis [29–31].

Resolution of ketoacidosis is considered when pH levels are > 7.3 and ketones below 0.3 mmol/L; insulin therapy and fluid resuscitation can be discontinued once the patient is eating and drinking and ketones are less than 0.6 mmol with normal bicarbonate.

Fetal wellbeing with continuous CTG monitoring is advocated as ketones readily cross the placenta and can severely compromise the fetus. The fetal mortality in diabetic and severe non-diabetic ketoacidosis is estimated at 9–35% with recent data suggesting 15.6% with advancements in diabetes care [32, 33]. CTG abnormalities are often corrected once maternal biochemical parameters normalise, therefore delivery is not always indicated [28]. Emergency operative delivery should be used with caution, as maternal condition can worsen if not adequately resuscitated and can result in an iatrogenic preterm birth. However, persistent maternal and fetal concerns require a multidisciplinary approach with escalation to critical care (Fig. 2) and consideration of early delivery, as delivery can lead to symptom resolution due to the removal of the fetus and placenta with a reduction in pregnancy-related hormones which oppose endogenous insulin [16].

Most pregnant women with SARS-CoV-2 are asymptomatic or have mild COVID-19. COVID-19 with concomitant euglycaemic ketoacidosis is rare, but should be considered in those who are unwell, have loss of appetite, and/or have gestational or Type 1/ Type 2 diabetes. Patient education about maintaining oral intake and glycaemic control during illnesses is paramount. Prompt recognition and timely management with multidisciplinary input are key to reducing the risk of adverse maternal and fetal outcomes.

## Patient perspective

At follow up, both patients were debriefed. Case 1 had had telephone monitoring at home with individual caseload midwifery and joint Endocrine/ Obstetric Medicine team prior to admission. She attended hospital immediately when her breathing deteriorated. She was aware her insulin intake had been suboptimal due to lack of appetite. Earlier admission may have prevented such severe metabolic acidosis. She was aware that on admission CTG was profoundly abnormal which could have led to an intrauterine death. Case 2 was surprised at the extent of metabolic derangement from starvation, having presented without any typical features of COVID-19.

## Data Availability

All relevant data described in the manuscript will be made available form the corresponding author upon request.

## Abbreviations

EDKA: Euglycaemic diabetic ketoacidosis
EKA: Euglycaemic ketoacidosis
DKA: Diabetic ketoacidosis
ARDS: Acute respiratory distress syndrome
BMI: Body mass index
CRP: C reactive protein
LDH: Lactate dehydrogenase
CTG: Cardiotocography
CTP: Computed tomography pulmonary angiogram
ACE-2: Angiotensin converting enzyme 2
RAAS: Renin–angiotensin–aldosterone system
